# Application of Carbon Nanotube-Based Elastomeric Matrix for Capacitive Sensing in Diabetic Foot Orthotics

**DOI:** 10.3390/mi16070804

**Published:** 2025-07-11

**Authors:** Monisha Elumalai, Andre Childs, Samantha Williams, Gabriel Arguello, Emily Martinez, Alaina Easterling, Dawn San Luis, Swaminathan Rajaraman, Charles M. Didier

**Affiliations:** 1 Department of Materials Science and Engineering, NanoScience Technology Center, University of Central Florida, Orlando, FL 32816, USA; monisha.elumalai@ucf.edu (M.E.); andre.childs@ucf.edu (A.C.); samantha.williams@ucf.edu (S.W.); ga150673@ucf.edu (G.A.); em422358@ucf.edu (E.M.); al433717@ucf.edu (A.E.); da125747@ucf.edu (D.S.L.); 2 Department of Electrical and Computer Engineering, Biomedical Engineering Program, and Burnett School of Biomedical Sciences, University of Central Florida, Orlando, FL 32816, USA; 3 Orthomerica Products Inc., Orlando, FL 32810, USA

**Keywords:** carbon nanotubes, elastomeric matrix, capacitive sensing, wearable sensors, orthotic sensors, diabetic foot ulcer offloading

## Abstract

Diabetic foot ulcers (DFUs) represent a critical global health issue, necessitating the development of advanced smart, flexible, and wearable sensors for continuous monitoring that are reimbursable within foot orthotics. This study presents the design and characterization of a pressure sensor implemented into a shoe insole to monitor diabetic wound pressures, emphasizing the need for a high sensitivity, durability under cyclic mechanical loading, and a rapid response time. This investigation focuses on the electrical and mechanical properties of carbon nanotube (CNT) composites utilizing Ecoflex and polydimethylsiloxane (PDMS). Morphological characterization was conducted using Transmission Electron Microscopy (TEM), Laser Confocal Microscopy, and Scanning Electron Microscopy (SEM). The electrical and mechanical properties of the CNT/Ecoflex- and the CNT/PDMS-based sensor composites were then investigated. CNT/Ecoflex was then further evaluated due to its lower variability performance between cycles at the same pressure, as well as its consistently higher capacitance values across all trials in comparison to CNT/PDMS. The CNT/Ecoflex composite sensor showed a high sensitivity (2.38 to 3.40 kPa^−1^) over a pressure sensing range of 0 to 68.95 kPa. The sensor’s stability was further assessed under applied pressures simulating human weight. A custom insole prototype, incorporating 12 CNT/Ecoflex elastomeric matrix-based sensors (as an example) distributed across the metatarsal heads, midfoot, and heel regions, was developed and characterized. Capacitance measurements, ranging from 0.25 pF to 60 pF, were obtained across N = 3 feasibility trials, demonstrating the sensor’s response to varying pressure conditions linked to different body weights. These results highlight the potential of this flexible insole prototype for precise and real-time plantar surface monitoring, offering an approachable avenue for a challenging diabetic orthotics application.

## 1. Introduction

Diabetes impacts 38.4 million people in the United States alone. This number accounts for approximately 11.6% of the total population [1] and is further exacerbated by diabetic neuropathy, leading to the loss of sensation in the extremities [2,3]. For many of these patients, foot ulcers may form (commonly referred to as diabetic foot ulcers or DFUs) and begin to break down the tissue in the lower extremities. In conservative numbers, it is estimated that DFUs affect a minimum of 5.7–9.5 million patients in a given year, where 1.5 million people are newly diagnosed annually [1,4,5]. In severe instances, limb amputation will result from such ulcers (approximately 19% of the total number of DFU patients) [6,7]. DFUs generally have a prolonged healing time, averaging 78 days, and a significant recurrence rate, with 40–65% of patients experiencing a recurrence within a year of healing [4,6]. Proper off-loading, which relieves pressure from the ulcer site, is essential for promoting effective treatment [8], while detecting early signs of foot ulceration is the most effective preventive strategy [3,9,10,11,12,13]. Non-invasive methods are available for DFU assessment, including laser Doppler flowmetry (which enables microvascular perfusion measurement for tissue viability evaluation); infrared thermography (which facilitates early inflammatory process detection through thermal mapping); plantar pressure and gradient analysis systems (which identify anatomically specific regions of mechanical stress); and ultrasound elastography (which provides a quantitative assessment of plantar tissue biomechanical properties) [14]. However, these methods lack availability as technological solutions in the reimbursable marketplace [15,16,17], limiting the capability for interventional treatments prior to serious complications [18].

In situ sensors, having diverse applications across various industries, are thus an attractive target for development and inclusion in diabetic orthotics. Across a variety of healthcare settings, wearable sensors are already utilized in situations such as for heart rate monitors to track a patient’s breathing rate and other vital signs [19,20], blood glucose level monitoring for diabetes management [21], continuous blood pressure monitoring [22], and biosensors for rapid disease detection [23]. Recent advancements in sensor technology encompass miniaturization, enhanced sensitivity, integration with IoT and edge systems, and the creation of smart sensors capable of real-time data processing and communication [24].

A pressure monitoring sensor system that helps prevent conditions that lead to ulcers offers significant advantages in diabetic monitoring applications, including early detection, dangerous pressure patterns leading to tissue damage followed by ulcer formation, complication prevention, pressure monitoring, reduced healthcare costs, and enhanced self-management, contributing to an improved quality of life. Leveraging IoT and edge technologies, these diabetic boots can enable remote monitoring for clinicians, patients, and researchers. Additionally, the pressure sensor can identify early indicators of ulcer formation, enabling timely intervention to prevent complications [25]. Pressure sensors used in this setting are primarily used for repetitive loading and unloading [26].

The conventional pressure and strain sensors typically applied in DFU diagnosis are based on semiconductors and metals, which exhibit limitations in stretchability and sensitivity. Their brittleness and poor elastomeric compatibility hinder applications in flexible sensing [27]. To overcome these limitations, there is an increasing demand to use novel materials-based nanoparticles [28], nanowires [29], graphene [30], and carbon nanotubes (CNTs) [31,32,33]. Recent advances have positioned CNTs as a promising material for next-generation flexible sensing technologies. Their unique mechanical and electrical properties offer significant potential in overcoming traditional sensor limitations [34]. CNTs along with polymer materials including PDMS and Ecoflex make an excellent choice for a composite, elastomeric matrix material for flexible sensors for wearable applications [35].

The development stages of the sensor were defined based on the type of sensor that would be fabricated, such as piezoresistive or capacitive, within the compositional and manufacturing limitations. An analysis of material sets covers those used in conventional strain sensors for non-biomedical applications, as well as those that are currently being researched due to their favorable properties for various applications, including biomedical and wearable technology [36]. This approach was guided by essential criteria to ensure that the sensor meets professional and societal standards including scalability, conformability, flexibility, biocompatibility, resistivity, durability, and the ability to withstand temperature and humidity variations [37,38,39,40]. Our work will be focusing on piezo-capacitive sensors. Pressure-capacitive sensors offer notable flexibility and cost-effectiveness in manufacturing [41].

Custom orthoses, designed using individualized patient data, offer a personalized fit that significantly improves pressure distribution and patient comfort. These devices have been shown to reduce plantar pressure and accelerate ulcer healing, particularly in patients with complex foot deformities or recurrent diabetic foot ulcers (DFUs) [42,43]. Although emerging technologies such as pressure-sensing insoles or smart footwear exist, they are not reliably covered by current reimbursement structures [11,44,45,46,47,48,49]. This gap underscores the broader systemic issue in diabetic wound care, whereby despite the availability of promising technologies, their adoption is constrained by limitations in healthcare policy and clinical reimbursement.

Therefore, there is a compelling need for research into pressure-sensing platforms that are not only clinically effective but that are also designed to align with existing healthcare reimbursement frameworks. Integrating sensor arrays into patient-specific orthoses offers a promising approach to improve clinical outcomes and enhance the monitoring of patient compliance. Such innovations hold the potential to overcome both clinical and socioeconomic barriers in DFU management [50,51].

In this work, we present the novel design of strain and pressure sensing materials for use in a sensor insole for orthotic boots. The parameters focus on designing an insole that can be laminated into the sole of the boot and can report and indicate where abnormal stress/pressure is occurring due to ulcer formation, leading to the prevention of diabetic foot ulcers. This work aims to begin tailoring the development of sensor-integrated, reimbursable, patient-specific orthotic insoles, addressing a critical gap in DFU management and enabling more equitable access to advanced wound care technologies.

## 2. Materials and Methods

### 2.1. Materials

Multiwall carbon nanotubes (MWCNTs), 70% isopropyl alcohol (IPA), and polydimethylsiloxane (PDMS) were purchased from Sigma Aldrich (St. Louis, MO, USA). Ecoflex 0030 (part A and B) was purchased from Smooth-On, Inc. (Macungie, PA, USA). PMMA acrylic substrates were purchased from Glassforge (Grants Pass, OR, USA). Lacey carbon grids (300 mesh, copper, with an approximate hole size of 63 µm) from Ted Pella Inc. (Redding, CA, USA) were purchased through Fisher Scientific (Waltham, MA, USA). Single-side adhesive Kapton sheets were purchased from CS Hyde (Lake Villa, IL, USA). The capacitance tester (MF470) and copper tape were purchased from Amazon (Seattle, WA, USA). Contact wires were purchased from Guangdong Rizhan High Temperature Wire Co., Ltd. (Gaozhan, China). Crepe foams were provided by Orthomerica Products Inc. (Orlando, FL, USA). Silver paste was purchased from AiTechnology (Princeton Junction, NJ, USA).

### 2.2. Methods

#### CNT Composite Synthesis

**Figure 1:**
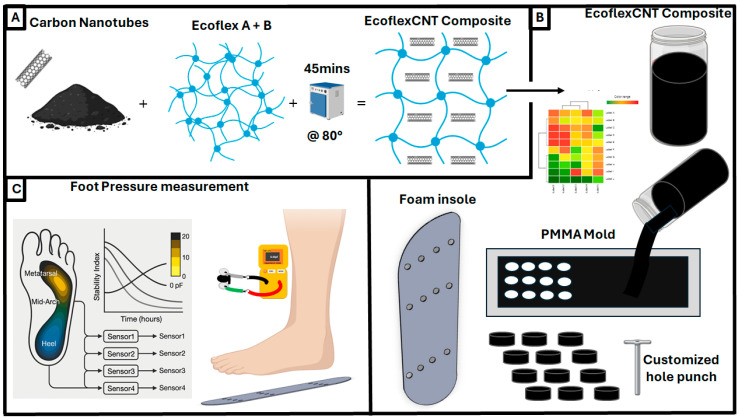
Schematic representation of a boot prototype for diabetic ulcer monitoring: (**A**) CNT/Ecoflex composite synthesis; (**B**) CNT/Ecoflex composite mixture; (**C**) the CNT/Ecoflex composite was cast on a larger acrylic mold (80 mm × 120 mm) to develop a larger set of sensor materials customized by a hole punch for the feasibility study (**right**) followed by an evaluation of stability and feasibility measurements (**left**).

The carbon nanotubes (CNTs) were incorporated (Figure 1 and Figure S1) into the polymer matrix by initially dispersing 0.06 g of CNTs in varying volumes of isopropyl alcohol (IPA), followed by sonication for 15 min [52]. The resulting dispersion was then thoroughly mixed with PDMS (10:1 ratio of the elastomer base and curing agent) or with 2 g of Ecoflex (Part A and Part B in a 1:1 ratio), as depicted in Figure S1, to obtain a homogeneous mixture. This yielded a final CNT weight percentage of 3 wt%. The effects of different IPA volumes (0, 2.5, 5, 10, and 15 mL) on the homogeneity of the mixture were systematically evaluated, along with the impact of sonication at various stages of the process. The uncured CNT composite was subsequently cast on prototypes designed in a PMMA acrylic mold of two sizes (20 × 60 mm and 80 × 120 mm) with a thickness of 1.1 mm. These molds were laser micromachined using a CO_2_ laser. The composite material was cured at 80 °C for 45 min. The sensor design is critical for optimizing pressure measurements and gauge factors. This optimization involves adjusting the ratio of conductive to elastomeric materials. Soft materials, such as elastomers, provide the necessary stretchability without plastic deformation, while preserving conductivity [53]. Incorporating conductive nanomaterials allows for the development of flexible conductors that combine the elastic properties of soft materials with high conductivity, which is suitable for biomedical applications. There is enough surrounding research to determine suitable pairs of conductors and soft plastics that have been used for other wearable sensor approaches [54,55]. For example, carbon nanotubes (CNTs) and polyvinylidene fluoride were previously mixed for the use of a piezoresistive sensor capable of detecting pulses with a gauge factor (GF) between 0.4 and 2.8 depending on the bending distance [56], whereas a CNT with thermoplastic polyurethane sensor had a GF range of 1 to 1.425 [57]. Alternatively, a polydimethylsiloxane (PDMS) and silver nanowire (AgNW) piezoresistive sensor was prepared through drop casting, with the ability to detect finger flexion with a GF between 2 and 20 depending on the percent of stretchability [58]. Based on these examples and additional research, our work in this paper formulates optimal mixtures of conductive and elastic materials, employing appropriate ratios and techniques to effectively design the boot sensor.

### 2.3. CNT and CNT Composite Characterization

**Figure 2:**
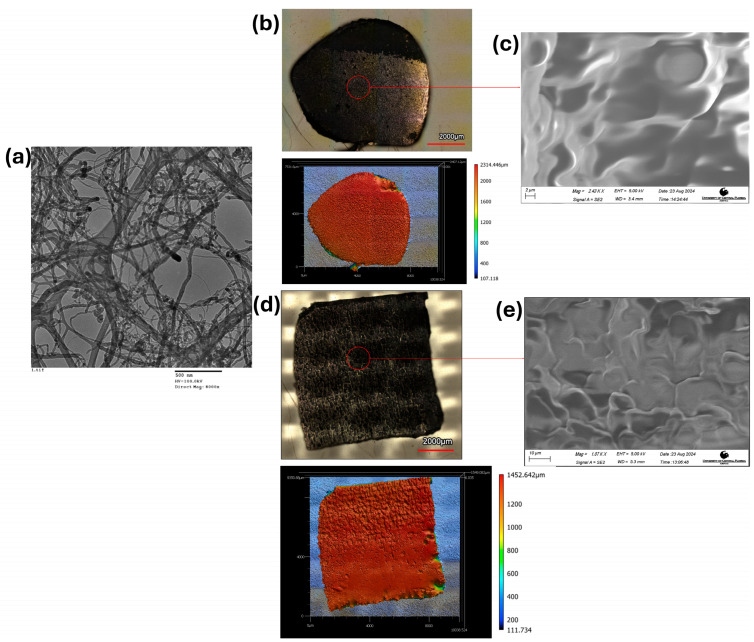
CNT and CNT/Ecoflex (3 wt% 1.1 mm thickness) characterization. (**a**) Transmission Electron Microscopy image of CNT; (**b**) Laser Confocal Microscopy image of cured CNT/Ecoflex (top part); (**c**) Scanning Electron Microscopy image of CNT/Ecoflex (top part); (**d**) Laser Confocal Microscopy image of cured CNT/Ecoflex (bottom part); (**e**) Scanning Electron Microscopy image of CNT/Ecoflex (bottom part).

A 5 µL aliquot of the CNT/IPA dispersion was deposited onto a copper grid, allowed to dry overnight, and characterized using Transmission Electron Microscopy (TEM) (JEOL TEM-1011, JEOL Ltd., Tokyo, Japan) with a 0.4 nm point-to-point resolution in order to investigate the structural, morphological, and chemical properties of the CNTs. Additionally, Scanning Electron Microscopy (SEM) (Zeiss Ultra 55 Field Emission Gun, Oberkochen, Germany) with a resolution of 1 nm @ 15 KV and 1.7 nm @ 1 KV was employed to perform an in-depth analysis of the surface morphology and distribution of CNTs within the polymer (PDMS/Ecoflex) matrix (5 mm × 5 mm, 3 wt%, and 1.1 mm of composite thickness with a gold layer deposited). The integrated SEM and TEM analyses offer a comprehensive understanding of CNT alignment, dispersion quality, and their interaction with Ecoflex, which are essential for optimizing the composite’s mechanical and electrical properties. Furthermore, Laser Confocal Microscopy (Surface Profiler (3D)–Keyence VK-X3000, Itasca, IL, USA) was used to analyze the texture of the CNT composites (3 wt% and 1.1 mm of composite thickness), providing detailed insights into the surface topography and internal structures at the micro- to nanometer scale, as observed in Figure 2 and Figure S2.

### 2.4. Electrical, Mechanical, and Stability Analysis of CNT Composite Sensors

The electrical properties of the materials were initially tested by cutting a 10 mm × 10 mm square sample. This sample was subsequently probed at opposite ends to monitor resistance changes under varying pressures applied by a thumb. CNT composites (PDMS/Ecoflex) were tested for their capacitance response at various weight percentages (1, 3, 5, and 10 wt%) with a constant thickness of 1.1 mm, as detailed in the supplementary information Tables S1–S4. The CO_2_ laser-fabricated PMMA molds with varying thicknesses (500 µm, 700 µm, 1.1 mm, and 1.5 mm) were evaluated, with the 1.1 mm depth yielding the most consistent and reliable results. Molds shallower than 1 mm posed sample removal challenges and frequent unstable measurements, whereas depths exceeding 1 mm introduced pores and bubbles, adversely affecting capacitive measurement stability. Pressure was applied in two ways—first qualitatively using the thumb and secondly, a commercial pressure sensing system (PressureGuardian^®^; Tillges, Maplewood, MN, USA) was used to provide quantifiable pressure measurements in pounds-per-square-inch (psi).

A maximum pressure of 60 psi was selected, representing the estimated pressure exerted by a 9 pound weight equivalent to thumb pressure. Five pressure intervals, each of 12 psi, were chosen to assess the material’s behavior across the range from 0 to 60 psi.

**Figure 3:**
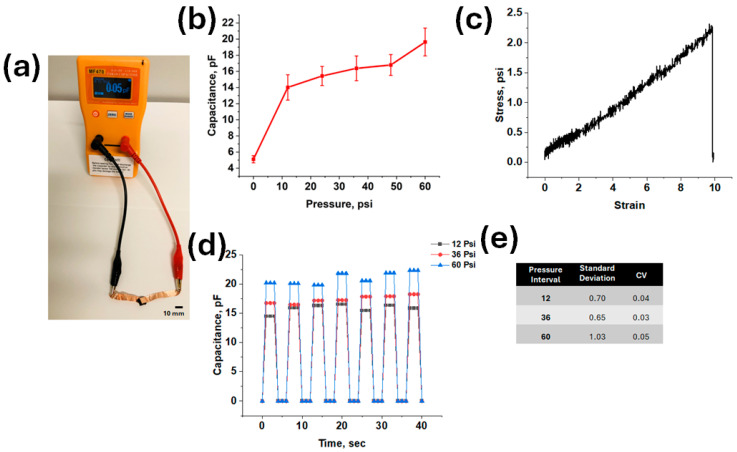
Electrical and mechanical properties of the 3 wt% CNT/Ecoflex composite material (1.1 mm thickness). (**a**) Capacitance testing using applied hand pressure on 3 wt% CNT/Ecoflex; (**b**) capacitance vs. pressure measurements on 3 wt% CNT/Ecoflex; (**c**) mechanical testing properties—tensile stress vs. strain of 3 wt% CNT/Ecoflex; (**d**) mechanical testing properties—cyclic testing of 3 wt% CNT/Ecoflex; (**e**) statistical data for cyclic testing of 3 wt% CNT/Ecoflex.

For clarity, the selected pressure intervals were also converted to kilopascals (kPa). To evaluate the suitability of the material for use in a walking/standing gait cycle, mechanical tests were conducted to determine its performance in an orthotic boot. Tensile testing and cyclic loading were performed on both PDMS/CNT (Figure S3) and Ecoflex/CNT (Figure 3) composites to quantify various behaviors and material characteristics. A tensile test was conducted using an Instron system (Instron 5969 universal testing machine, MA) to evaluate the key mechanical properties of the composite polymers. The primary objective was to determine Young’s Modulus, which quantifies the material’s resistance to elastic deformation under stress. Maximum strain is directly recorded from the Instron system. The tensile testing procedure for these elastomers followed ASTM D412, which is the standard for thermoplastic elastomers. ASTM D412 specifies 5 kN pneumatic grips, which were utilized with a maximum load of 25 kN each, reducing experimental sensitivity and introducing noise into the tensile testing graphs [59]. The standard’s elongation rate of 500 ± 50 mm/min was converted to inches per minute. The ASTM D412 test was conducted with the materials cut to three coupons per material, with average dimensions of 65 × 10 × 5 mm for larger samples.

### 2.5. Prototype Design

A foam insole (38.1 mm thickness) from Orthomerica was precisely cut to a length corresponding to ten inches from a sheet of Sekisui Voltek VOLARA TYPE EO [60]. The sensors for the prototype were fabricated as outlined in Methods Section 2.1 of this paper. The insole was covered by a single-side adhesive Kapton sheet (0.05 mm thickness), followed by attaching a wire to each sensor using silver paste. The foam material (31.8 × 82.6 × 2.29 mm) was cut into a rectangular shape, fixed onto the Kapton sheet, and the 8 mm diameter CNT/Ecoflex sensors were strategically integrated into the insole within the foam material, in two different protypes. One had a single sensor (total size of prototype: 31.8 × 50.8 mm), whereas the other had 12 sensors and was used as a demonstratable example sensor array metatarsal (31.8 × 82.6 mm), mid arch (44.5 × 63.5 mm), and heel (44.5 × 69.9 mm). The process is highly customizable for higher-density sensor arrays and individualization depending on the patient in question. A total of four insole sensor array prototypes were prepared, where each prototype consists of three sensors placed in the metatarsal, mid arch, and heel points of the insole, covering key regions of the feet in order to monitor the pressure, totaling twelve sensors. Each insole sensor array prototype was connected to three capacitance meters for capacitance monitoring, as observed in the supplementary info (Figures S4 and S5), followed by the application of silver paste. Wires were then attached, and the assembly was placed in an oven to cure for one hour, as depicted in Figure 1B. Following the curing process of the silver paste, the prepared insole was inserted into a boot from Orthomerica. The wires were extended through the boot, which had been modified using a drill, to ensure a seamless connection and functionality, as observed in the supplementary info (Figure S4b). The capacitance of the sensor under an applied pressure from a human foot was recorded using a capacitance meter, as illustrated in Figure 1C.

### 2.6. Heat Mapping—Matlab/Python

The pressure sensing data, utilized for the heat mapping analysis of the cured sensor material layout, were analyzed to identify areas with consistent data. This analysis, along with data from multisensor arrays used in a feasibility study, was prepared in Jupyter Notebooks using Python (version 3.11.10) code. The Python scripts, developed with the matplotlib and numpy packages, facilitated the processing and visualization of the pressure data from the developed sensors and volunteer test data, ensuring accurate and detailed heatmaps. In order to evaluate the practicality of the testing protocol, measurements were conducted using three volunteers with varying physical profiles to simulate a range of potential end-user conditions. The test included one male and two female participants, representing different foot sizes (6.5, 7, and 12 inches) and body weights (124, 175, and 176 pounds). Pressure sensing data at the metatarsal, mid-arch, and heel regions for three participants were analyzed using heat mapping. Each participant’s results were displayed side by side to assess consistency and usability across diverse user profiles. This approach allowed for the separation in the display of live data processing and visualization through the heat mapping of pressure locations, as well as providing insights into sensor performance and reliability. Furthermore, a detailed analysis is described in Section 3.

## 3. Results and Discussion

Developing pressure sensors for orthotic boots involves complex developmental considerations with significant cost implications [61]. Material selection is crucial, focusing on conductive and durable components that are essential for sensor function. In our work, we utilized a CNT polymer composite to address the dual need for flexibility and conductivity for a wearable sensor [62]. Two types of CNT composites were prepared—CNT/PDMS and CNT/Ecoflex. Detailed information on CNT/PDMS synthesis is provided in the Supplementary Information (Figure S1a). Figure S2b shows a detailed description of CNT/Ecoflex composite synthesis. Different IPA volumes resulted in less homogeneity in the CNT composite, as well as bubbles on the sample’s surface after curing [41]. The optical figures show the uniformity of the composite with different volumes of IPA before and after curing (Figure S1c,d). Thus, the CNT composite with 0% IPA was selected for further sensor development. The carbon nanotube material was characterized using TEM [63], revealing its morphology, which shows long, cylindrical, and hollow tube-like structures with a diameter of 110–170 nm and a length of 5–9 µm, as shown in Figure 2a. Figure 2b,d depict Laser Confocal Microscopy images of the CNT/Ecoflex material (top and bottom views) post-curing, revealing a porous structure that contributes to enhanced sensitivity, while Figure 2c,e show SEM characterization of porous and evenly distributed CNTs in the Ecoflex matrix, which is significant for the overall sensitivity of the sensor material. The varying weight percentages (1, 3, and 5 wt%) and thicknesses (500 µm, 700 µm, 1.1 mm, and 1.5 mm) of the CNT composite significantly influenced the capacitance measurements, as detailed in the supplementary information.

The 3 wt% CNT/Ecoflex composite with a 1.1 mm thickness was selected for further experiments due to its wide range of capacitance, better mechanical properties, and greater reliability. The capacitance measurement showed an increase in values recorded based on input pressures. The average (N = 3) results of CNT/Ecoflex were compared with CNT/PDMS, as detailed in supplementary information Figure S3. The results shown in Figure 3b indicate that it exhibits slightly higher capacitance (~2 pF) values when compared to CNT/PDMS. However, due to their similar performance, neither composite could be definitively favored without considering the full application. Further mechanical and fatigue testing were conducted to evaluate material behavior under application-specific conditions [64]. Although the electrical properties of the composite materials are crucial for the application, the mechanical properties are equally vital for the success of the composite as an orthotic insole. Given its use in a walking/standing gait cycle, the tensile strength and cyclic loading tests on both the CNT/PDMS and CNT/Ecoflex composites were conducted to evaluate their suitability for repeated use [65]. The mechanical property testing of CNT/PDMS is detailed in supplementary information Figure S3. Softer materials, such as elastomers, exhibit low Young’s moduli, while most metals and carbon nanotubes have exceptionally high values. Pure PDMS typically exhibits Young’s modulus ranging from 1.32 to 2.97 MPa, whereas pure Ecoflex has a reported modulus of 125 kPa [66,67]. The significant difference in Young’s moduli between these two polymers aligns with Ecoflex’s greater flexibility and lower stiffness compared to PDMS when cured [68]. The incorporation of CNTs into the polymers is expected to potentially enhance the composite’s modulus, given that CNTs by themselves possess an extremely high modulus range of 270–950 GPa [69]. However, the extent of this increase is modest and depends on factors such as CNT orientation and concentration [70]. Tensile testing not only determines Young’s modulus but also measures the maximum strain and ultimate yield strength of the composite polymers. Given the nonlinear elastic behavior of elastomers, we focus on ultimate yield strength, which indicates the maximum load that the material can sustain while deforming plastically.

The tensile test output, initially reported as load versus displacement, was subsequently converted to stress and strain values. After converting the raw data into stress (psi) and strain (%) values, the graph can be plotted and analyzed. The tensile test results for 3 wt% Ecoflex/CNT are analyzed, with the stress vs. strain graph being presented in Figure 3c. The Ecoflex-based composite exhibits the behavior characteristics of an ideal elastomer, showing a nonlinear elastic region and a gradual increase in stress until failure. The material fails only at a stress value of 2.25 psi and a strain of 9.98%, while the stiffness and Young’s modulus of the material are still under investigation.

Comparing the composites reveals trade-offs. PDMS/CNT has a higher preliminary Young modulus but a lower strain tolerance, while Ecoflex/CNT endures greater strain but risks plastic deformation due to its lower preliminary modulus [68,71]. Cyclic loading tests, which are essential for assessing real-world application and durability, were conducted at three pressure levels (12 psi, 36 psi, and 60 psi) using new samples (10 × 10 mm), with capacitance being recorded using the PressureGuardian^®^, at 3 s intervals; the results are shown in Figure 3d and Figure S3c. The coefficient of variation and standard deviation calculated for each sample provided insights into the reliability and performance of the materials under cyclic loads, with detailed data being presented in Figure 3e.

Comparing the cyclic loading results of CNT/Ecoflex and CNT/PDMS, CNT/PDMS exhibits greater fluctuations than CNT/Ecoflex. Generally, the standard deviation and coefficient of variation (CV) are higher for CNT/PDMS, indicating reduced reliability under cyclic loads, particularly at lower pressures. However, CNT/PDMS shows a greater range of capacitance values between pressure intervals, reflecting a higher sensitivity compared to CNT/Ecoflex [72]. Conversely, CNT/Ecoflex demonstrates a lower variability between cycles at the same pressure, as well as consistently higher capacitance values across all trials. Thus, the CNT/Ecoflex composite material was selected for further experiments. The pressure sensor was created and tested for the purpose of measuring different capacitive values and comparing pressure points on the foot during regular locomotion. This will provide information on the location and magnitude of pressure during steps for test volunteers. The sensitivity of the CNT/Ecoflex composite sensor showed a high sensitivity (2.38 to 3.40 kPa^−1^) over a pressure sensing range of 0 to 68.95 kPa.

**Figure 4:**
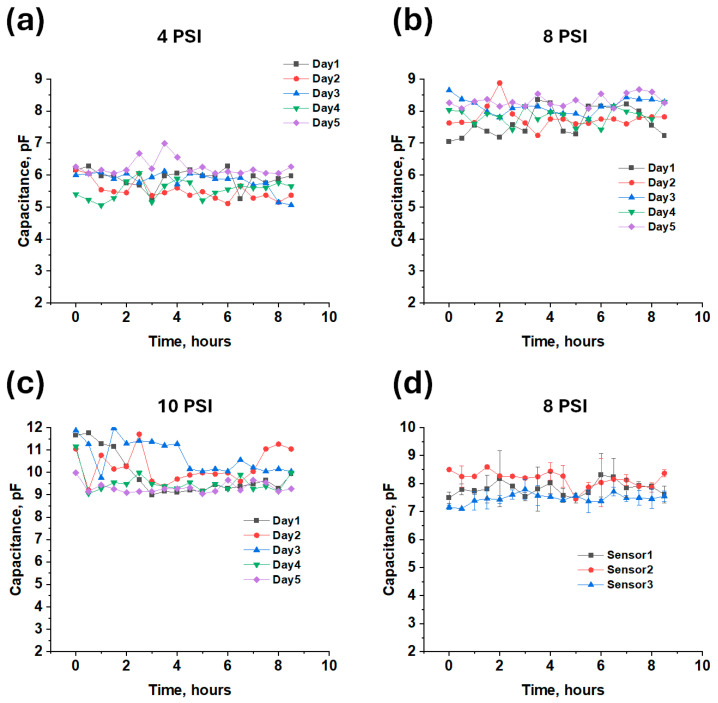
Stability evaluation of the CNT/Ecoflex sensor conducted over 30 min intervals, with a 1 min break between each, for a total duration of 8.5 h per day over 5 days. (**a**) CNT/Ecoflex at 4 PSI; (**b**) CNT/Ecoflex at 8 PSI; (**c**) CNT/Ecoflex at 10 PSI; (**d**) sensor-to-sensor variation at 8 PSI (n = 2).

Across an applied pressure of 4 psi for 5 days, the microsensor platform demonstrated stable capacitive responses, spanning a range of ~5.5 to 6.5 pF +/−1 pF (Figure 4a). An applied 8 psi pressure demonstrated a reliable stability between 7 and 8.5 pF +/−1.5 pF (Figure 4b). Finally, an applied 10 psi pressure demonstrated a response between 9 and 11.5 pF +/−2.5 pF, as shown in Figure 4c. Based on the preliminary stability test results, the high variability observed in the sensor measurements during the first three days under a 10 PSI load may be attributed to changes in sensor positioning. This variability appears to stabilize over time, as seen in the data from the fourth and fifth days. However, further testing is needed in future studies, with an increased number of test cycles being conducted in a controlled and automated environment. Sensor-to-sensor variation was also investigated at an 8 psi pressure. All three sensors that were tested were from the same synthesized batch and showed a capacitance between 7 and 8.5 pF within 1.5 pF variation, showing the sensor’s promising reliability, as shown in Figure 4d. Even though promising results are demonstrated from this work, more studies need to be conducted to reduce the variability to under 10% or lower.

### 3.1. CNT/Ecoflex Composite Fabrication

**Figure 5:**
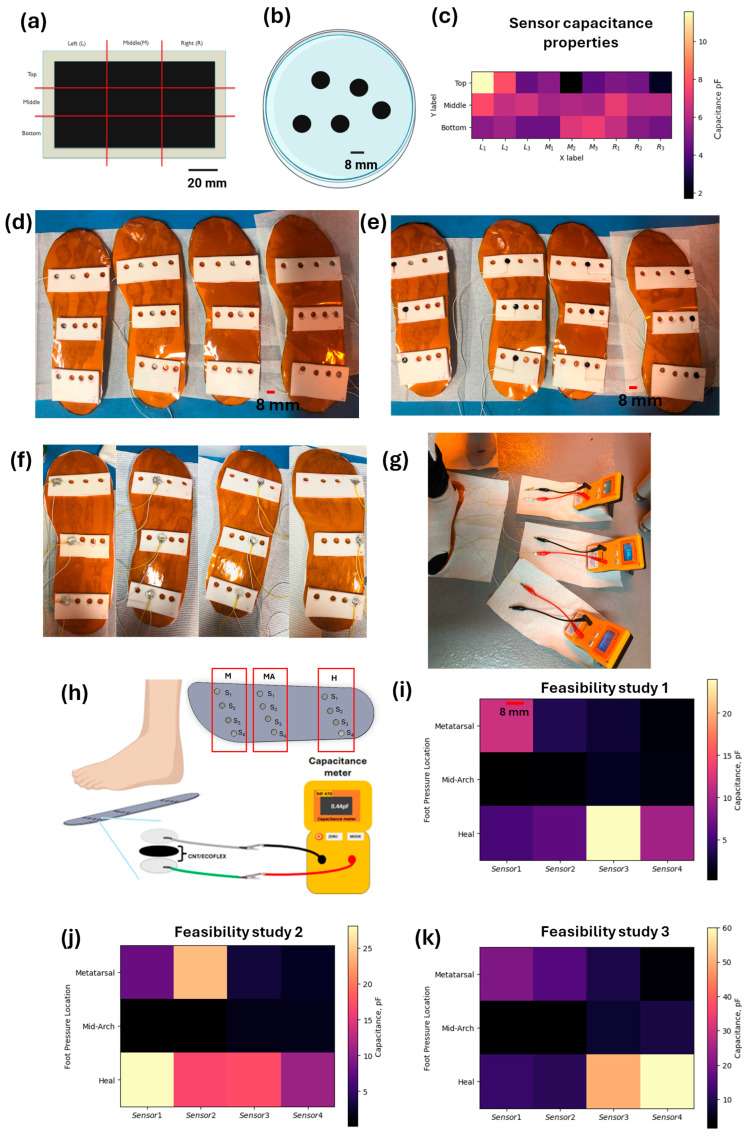
Implementation of a CNT composite sensor array with conductive silver traces on a Kapton sheet integrated into a shoe insole. (**a**) CNT/Ecoflex cured on an acrylic mold (80 mm × 120 mm); (**b**) 8 mm CNT/Ecoflex sensors; (**c**) evaluation of CNT/Ecoflex sensors’ capacitance measurements when cured on a mold using heat mapping data. The structural and integration of capacitive sensor into the insole. (**d**) Step1: Insole arrangement with 8 mm diameter hole with silver paste and bottom wire for conductivity; (**e**) insole arrangement with 8 mm diameter CNT/Ecoflex sensor placed within the hole; (**f**) silver paste with top wire placed above the sensor, forming a capacitive sensor setup; (**g**) feasibility study setup; (**h**) N = 3—feasibility study of CNT/Ecoflex sensor implemented into the insole with sensor 1 (S_1_), sensor 2 (S_2_), sensor 3 (S_3_), and sensor 4 (S_4_) on the metatarsal (M), midfoot (MA), and heel (H); (**i**) feasibility study 1 of 176 pounds (6.5 inch feet size); (**j**) feasibility study 2 of 124 pounds (6.5 inch feet size); (**k**) feasibility study 3 of 175 pounds (12 inch feet size).

The 3 wt% CNT/Ecoflex composite was cast on a larger acrylic mold (80 mm × 120 mm) to develop a larger set of sensor materials for the volunteer study and to test the repeatability of capacitive transduction throughout the experiment. After the CNT/Ecoflex material was cured, the material was cut into different sections, as shown in Figure 5a, followed by punching out the sensors in a circular shape of ~8 mm diameter, as shown in Figure 5b. The capacitance increase from sections of the mold can be observed in the heat map shown in Figure 5c. The best location for repeatable sensor performance is shown to be in the center of the mold, as expected. The structural design and integration of the capacitive sensor into the insole involved a multi-step process. Initially, an 8 mm diameter hole was created in the support; then, this was pasted above the insole and filled with silver paste along with a bottom wire to ensure conductivity, as shown in Figure 5d. A CNT/Ecoflex sensor of the same diameter was then placed within the hole, as shown in Figure 5e. To complete the capacitive sensor setup, silver paste and a top wire were applied above the sensor, as shown in Figure 5f. Finally, a feasibility study was conducted to evaluate the performance of the integrated sensor system, as shown in Figure 5g. The capacitance of the sensor under applied pressure from a human foot was recorded using a capacitance meter, as illustrated in Figure 5h. A three-volunteer feasibility study (one healthy male and two healthy females) was conducted to analyze the variation in sensitivity vs. pressure applied by each user.

The results of feasibility study 1, 2, and 3 (detailed below) showcase the pressure applied by an individual of 176 pounds (6.5 inch feet size), 124 pounds (6.5 inch feet size), and 175 pounds (12 inch feet size), respectively, as is depicted in Figure 5i–k.

#### 3.1.1. Feasibility Study 1

In feasibility study 1 (Figure 5i), the heat mapping data of the metatarsal (M), midarch (MA), and heel points (H) at metatarsal sensor points 1 and 2 showed higher capacitance values ranging from 7 to 12 pF, while M-S3 and S4 showed a capacitance lower than ~1 to 3 pF. Similarly, feasibility study 1 showed increasing capacitance values from MA S1 to S4, which justifies the fact that the pressure is less in MA-S1 compared to S2, S3, and S4; this is due to the arch position of the feet and is related to the contact with the sensor points. H-S3 and S4 showed a capacitance above 4 pF, while H-S1 and S2 points showed lower capacitance values.

#### 3.1.2. Feasibility Study 2

The heat mapping data of feasibility study 2 are shown in Figure 5j, where M-S1, S2, and S3 showed a capacitance range between 7.5 and 22.5 pF, while M-S4 showed a capacitance below 7.5 pF, demonstrating more pressure at points S1 to S3. Similarly, MA-S3 and S4 showed a higher capacitance compared to S1 and S2, followed by H, demonstrating a higher capacitance in points S1 and S4 compared to S2 and S3 based on the foot arch position placed on the insole.

#### 3.1.3. Feasibility Study 3

The heat mapping data of feasibility study 3, as shown in Figure 5k, demonstrated higher capacitance changes at M-S1 and S2 in comparison to S3 and S4, while the MA showed a high capacitance value in points S3 and S4, depicting capacitance in the range of 5 to 8 pF; the other two points showed a capacitance lower than 5 pF. The H points of the feasibility study showed a high capacitance in heel points S3 and S4 above 50 pF, demonstrating the high pressure applied in a manner that is directly proportional to capacitance changes. The prototype demonstrated consistent results across sensor outputs and varying applied pressures with individuals of different weights. Initially, thumb-applied hand pressure was used to evaluate the electrical and mechanical properties of the CNT composite material under a controlled pressure-sensing environment. This step was followed by applying foot pressure/natural movement cycles on an array of sensors integrated into a shoe insole, with sensor outputs being measured as capacitance values in order to quantitatively assess the overall performance of the sensor in different conditions.

This study initiates a pathway for developing a boot prototype for diabetic foot ulcer monitoring and designing a flexible circuit-based insole array as a future work for sensor array development and testing in DFU patients. The variations in plantar pressure distribution between healthy individuals and those with diabetic foot complications can potentially be identified and tracked to enable early intervention. The feasibility studies performed herein with healthy volunteers serve as an initial step towards the further development and eventual clinical validation of the sensing system, utilizing diabetic patient volunteers. It is again important to contextualize this work within the broader landscape of diabetic care, specifically in relation to orthotic bracing. While not a directly applicable technological solution at this stage, the results are encouraging as they demonstrate the preliminary discernment of differential pressures from healthy patient profiles.

Thus, the potential of CNT-based elastomeric matrix sensors for health-monitoring applications is promising for several reasons including stability, scalability, reliability, biocompatibility, and power consumption, not to mention providing avenues for realizing reimbursable diabetic monitoring solutions. However, within the scope of this work’s future directions, capacitive sensors are susceptible to environmental factors such as temperature and humidity. Given that the sensor is housed in a boot and is in direct contact with the body, effective encapsulation is necessary to mitigate the effects of perspiration and heat buildup, which can lead to increased humidity levels, subsequently guiding future directions of this work. Moreover, fabrication techniques are of great interest when considering a product that should be scalable; thus, understanding what has been and is currently being carried out to fabricate these sensors is crucial to develop an optimized method to create sensors for orthotic insoles that can reach a wide patient population, while retaining excellent performance properties and durability.

## 4. Conclusions

In summary, a capacitive pressure-sensitive material, utilizing flexible composites, was demonstrated as a novel, reimbursable fabrication for diabetic orthotics. The CNT and polymer composites were chosen based on their mechanical properties, electrical performance, and industry fabrication compatibility. CNT composites of 3 wt% were fabricated and cured using an acrylic mold with a final thickness of 1.1 mm for consistent performance, resulting in the ability to extract capacitance-based pressure metrics. The CNT/Ecoflex composite sensor showed a high sensitivity (2.38 to 3.40 kPa^−1^) over a pressure sensing range of 0 to 68.95 kPa. The sensor demonstrated excellent performance characteristics, with a capacitance-based monitoring system, showing consistent stability in capacitance (6.5, 8.5, 11.5 pF) across different pressure ranges (4, 8, and 10 psi). Sensor to sensor variability was tested at 8 psi; three sensors from the same batch exhibited a capacitance between 7 and 8.5 pF with only 1.5 pF variation, indicating promising reliability. The sensor’s successful integration into an Orthomerica OWLS diabetic orthosis prototype validated its practical application through the selected feasibility studies, highlighting its potential for improved diabetic foot care monitoring.

## Data Availability

The original contributions presented in this study are included in the article/Supplementary Materials. Further inquiries can be directed to the corresponding author.

## Supplementary Materials

The following supporting information can be downloaded at https://www.mdpi.com/article/10.3390/mi16070804/s1, Figure S1: (a) CNT/PDMS composite synthesis (b) CNT/Ecoflex composite synthesis (c) CNT/Ecoflex curing on mold synthesized with different IPA volumes (d) CNT/Ecoflex composite with different IPA volumes after curing; Figure S2: CNT/PDMS characterization (a) Laser confocal image top view (b) Laser confocal image bottom view (c) Scanning electron image of CNT/PDMS top view (d) Scanning electron image of bottom view; Figure S3: Electrical and mechanical properties of the 3 wt% CNT/PDMS (1.1 mm thickness) composite material (a) Capacitance vs pressure measurements using applied hand pressure on 3 wt% CNT/PDMS (1.1. mm thickness) (b) Mechanical testing properties Tensile stress vs strain; (c) Mechanical testing properties of 3 wt% CNT/PDMS (1.1. mm thickness) Cyclic testing (d) Statistical data for cyclic testing of 3 wt% CNT/PDMS (1.1 mm thickness); Figure S4: (a) Capacitance measurements of the sensor array in the CNT/Ecoflex sensor integrated into the insole boot prototype (b) Shows capacitance measurements of the sensor array in the CNT/Ecoflex sensor integrated into the insole boot prototype setup; Figure S5: Stability measurement setup for CNT/Ecoflex sensor; Table S1: Electrical properties of 1 wt% CNT/PDMS and CNT/Ecoflex sample capacitance data versus various qualitative pressures applied through fingers; Table S2: Electrical properties of 3 wt% CNT/PDMS and CNT/Ecoflex capacitance data versus various quantitative pressures applied through fingers using pressure guardian; Table S3: Electrical properties of 5 wt% CNT/Ecoflex sample capacitance data versus various qualitative pressures applied through fingers at two different temperatures (25 °C and 80 °C); Table S4 CNT composite weight percentage comparison and evaluation.

## Abbreviations

Diabetic foot ulcers (DFUs); carbon nanotube (CNT); polydimethylsiloxane (PDMS); Transmission Electron Microscopy (TEM); Scanning Electron Microscopy (SEM); isopropyl alcohol (IPA); coefficient of variation (CV).
