# Supplementary material for: Application of Carbon Nanotube-Based Elastomeric Matrix for Capacitive Sensing in Diabetic Foot Orthotics
Source: Micromachines (Basel). 2025 Jul 11;16(7):804. doi: 10.3390/mi16070804 (PMC12300393; doi:10.3390/mi16070804)
Supplement: Supplementary file 1 [file micromachines-16-00804-s001.zip › micromachines-3708198-supplementary.pdf]

Application of Carbon Nanotube-based Elastomeric Matrix for Capacitive Sensing in Diabetic Foot Orthotics

Monisha Elumalai[1], Andre Childs[1], Samantha Williams[1], Gabriel Arguello[1], Emily Martinez[1], Alaina Easterling[1], Dawn San Luis[1], Swaminathan Rajaraman[1,*], and Charles M. Didier[2,*]

1 University of Central Florida, Orlando, Florida, USA
2 Orthomerica Products Inc., Orlando, Florida, USA
* Correspondence: cdidier@orthomerica.com; swaminathan.rajaraman@ucf.edu

Experimental section

CNT/PDMS composite synthesis

The initial step involved accurately measuring the required amount of CNT powder, followed by dispersion in IPA, rather than preparing a stock solution. Following, the CNT-IPA mixture was subjected to sonication to enhance dissolution for 10 to 15 minutes. After sonication, the polymer was added to achieve the desired weight percentage of nanofiller. For PDMS, the elastomer base and curing agent were combined in a 10:1 ratio, added sequentially with final composition 3wt%. Further the CNT/PDMS composite was placed on a PMMA mold and subjected to heat at 80ºC for 45 min. The cured CNT/PDMS was used for testing electrical and mechanical properties.

CNT/PDMS or Ecoflex curing on PMMA mold

A reusable PMMA mold with varying thicknesses (500 µm, 700 µm 1.1 mm, 1.5 mm) was created using a CO_2 laser. Mold thickness 500 µm was either too thin or too brittle to be properly removed from the mold, leading to inconsistent samples and unreliable data.

CNT/PDMS composites ranging from 1 wt% to 10 wt% and found nominal conductivity gains beyond 5 wt%. To simplify synthesis and optimize for aspects such as cost-effectiveness, testing 1 wt% and 3 wt% composites was considered, ruling out other CNT-to-polymer ratios. The mold thickness (1.1 mm depth) produced ideal thickness with minimal complications, while eliminating mold thickness (500 µm, 700 µm and 1.5 mm).

Electrical and Mechanical properties of 1 wt% CNT/PDMS and CNT/Ecoflex

Further testing was carried out with a single standardized procedure, a fixed mold thickness, and three CNT weight ratios. Starting with 1 wt%, CNT/PDMS and CNT/Ecoflex composites were fabricated at a mold depth of 1.1 mm, and three trials were conducted. The raw data is presented in Table S1. The 1 wt% samples showed lower mechanical robustness and inconsistent capacitance values under similar loads, leading to concerns about their reliability and exclusion from further testing.

After excluding 1 wt% and 5 wt%, The 3 wt% CNT composites with both polymers electrical properties were tested. The thumb pressure to samples were quantified, in pounds-per-square-inch (psi)., using pressure guardian (PressureGuardian®;Tillges, USA) in pounds-per-square-inch (psi) by increments of 12 psi. The results for 3 wt% are presented in Table S2. This concentration was found to be optimal, showcasing responsiveness to pressure and more favorable mechanical properties compared to the 1 wt% and 5 wt% samples.

The 5 wt% CNT/Ecoflex composite showed a broader capacitance range under consistent loads as shown in Table S3. The difference in capacitance values between 3 wt% and 5 wt% was minimal. Considering efficient utilization of materials, 3 wt% CNT was preferred. Thus, 3 wt% was the optimal CNT-to-polymer ratio, offering a wide capacitance range/large sensing window, better mechanical properties, and greater reliability.

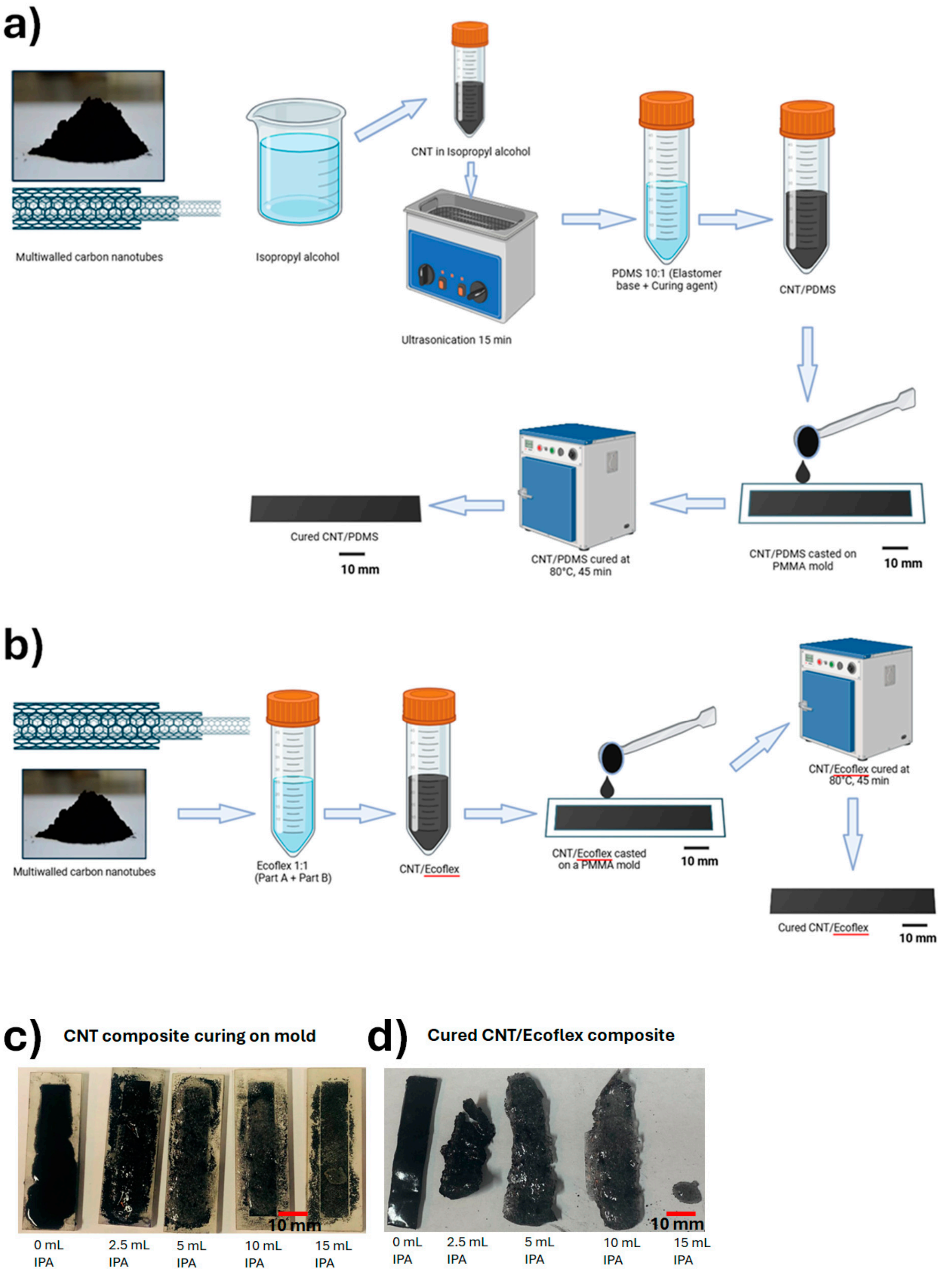

Figure S1 a) CNT/PDMS composite synthesis b) CNT/Ecoflex composite synthesis c) CNT/Ecoflex curing on mold synthesized with different IPA volumes d) CNT/Ecoflex composite with different IPA volumes after curing

CNT-PDMS characterization

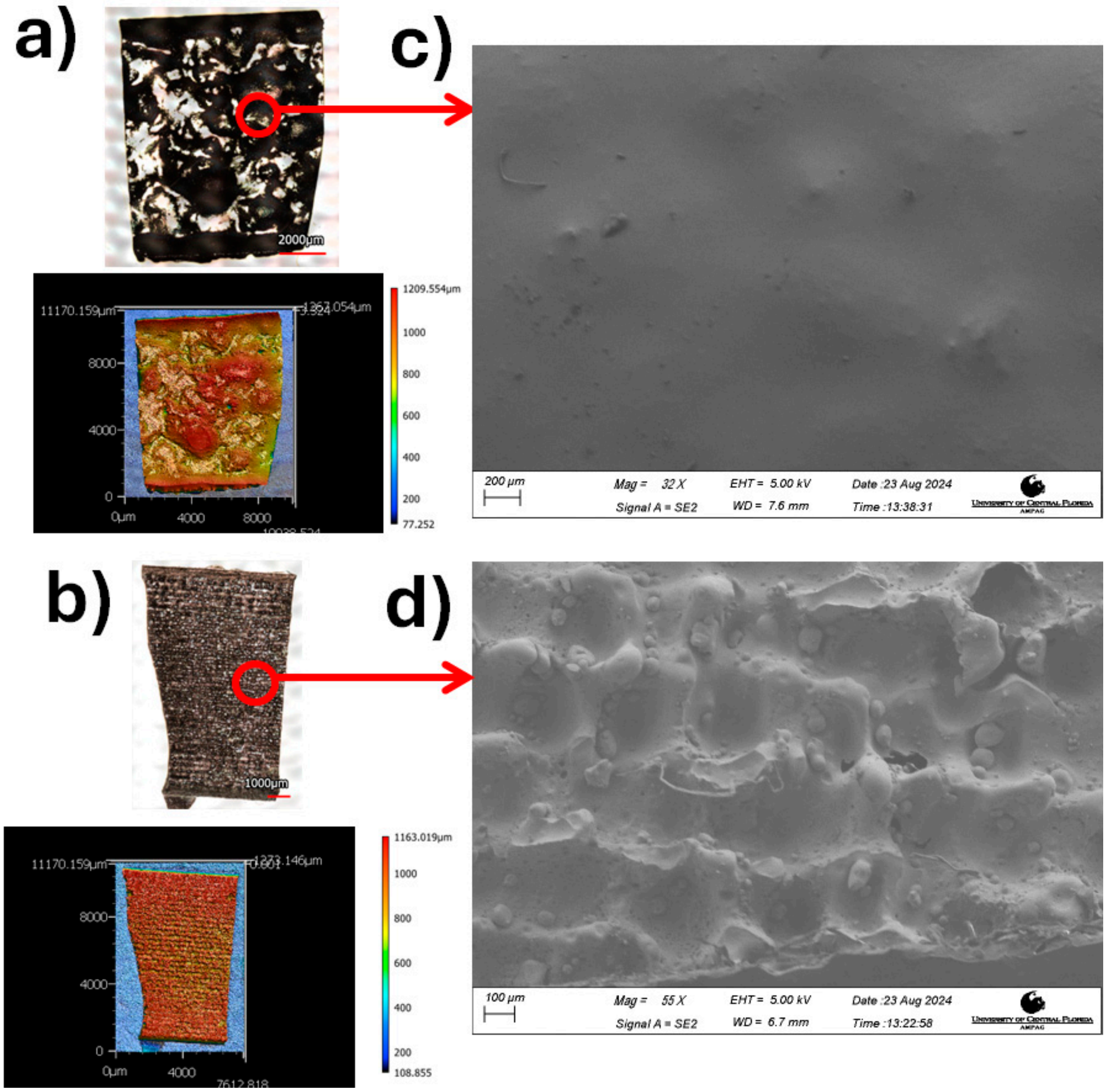

Figure S2 CNT/PDMS characterization a) Laser confocal image top view b) Laser confocal image bottom view c) Scanning electron image of CNT/PDMS top view d) Scanning electron image of bottom view

Table S1 Electrical properties of 1 wt% CNT/PDMS and CNT/Ecoflex sample capacitance data versus various qualitative pressures applied through fingers

1 wt% Samples	Initial (pF)	Light Pressure (pF)	Medium Pressure (pF)	Heavy Pressure (pF)
**CNT/PDMS**	9.26	17.86	20.25	24.34
	8.54	15.92	20.58	23.13
	8.93	12.48	18.36	19.08
**CNT/Ecoflex**	6.21	13.76	17.81	22.30
	7.10	12.48	13.81	15.64
	8.93	13.98	16.31	17.53

Table S2 Electrical properties of 3 wt% CNT/PDMS and CNT/Ecoflex capacitance data versus various quantitative pressures applied through fingers using pressure guardian

3 wt% Samples	0psi Capacitance (pF)	12psi Capacitance (pF)	24psi Capacitance (pF)	36psi Capacitance (pF)	48psi Capacitance (pF)	60psi Capacitance (pF)
**CNT/PDMS**	6.10	17.20	20.36	21.14	21.63	23.58
	8.65	16.64	17.25	19.14	20.47	22.58
	7.49	13.87	17.31	18.14	19.03	20.30
**CNT/Ecoflex**	10.37	18.97	21.86	23.13	24.35	26.35
	9.43	19.91	20.64	22.30	24.35	27.85
	10.04	13.81	21.52	23.47	26.07	28.07

Table S3 Electrical properties of 5 wt% CNT/Ecoflex sample capacitance data versus various qualitative pressures applied through fingers at two different temperatures (25ºC and 80 ºC)

Ecoflex Samples	Mold Depth (mm)	Initial (pF)	Light Pressure (pF)	Medium Pressure (pF)	Heavy Pressure (pF)
**5 wt% at 25 ºC**	0.7371	6.55	17.25	22.47	27.51
	1.1274	11.71	22.86	25.02	30.18
	1.5199	9.1	18.69	21.36	24.52
**5 wt% at 80 ºC**	0.7371	9.65	22.3	25.19	27.13
	1.1274	10.6	21.47	25.13	31.73
	1.5199	10.15	17.81	22.86	29.46

Table S4 CNT composite weight percentage comparison and evaluation

Observed measurements	1%wt.	3%wt.	5%wt.	8%wt.	10%wt.
**Slow climb**	✕	✕	✕		
**OL Error**	✕			✕	✕
**Inconsistency**	✕			✕	✕
**High C_0**				✕	✕

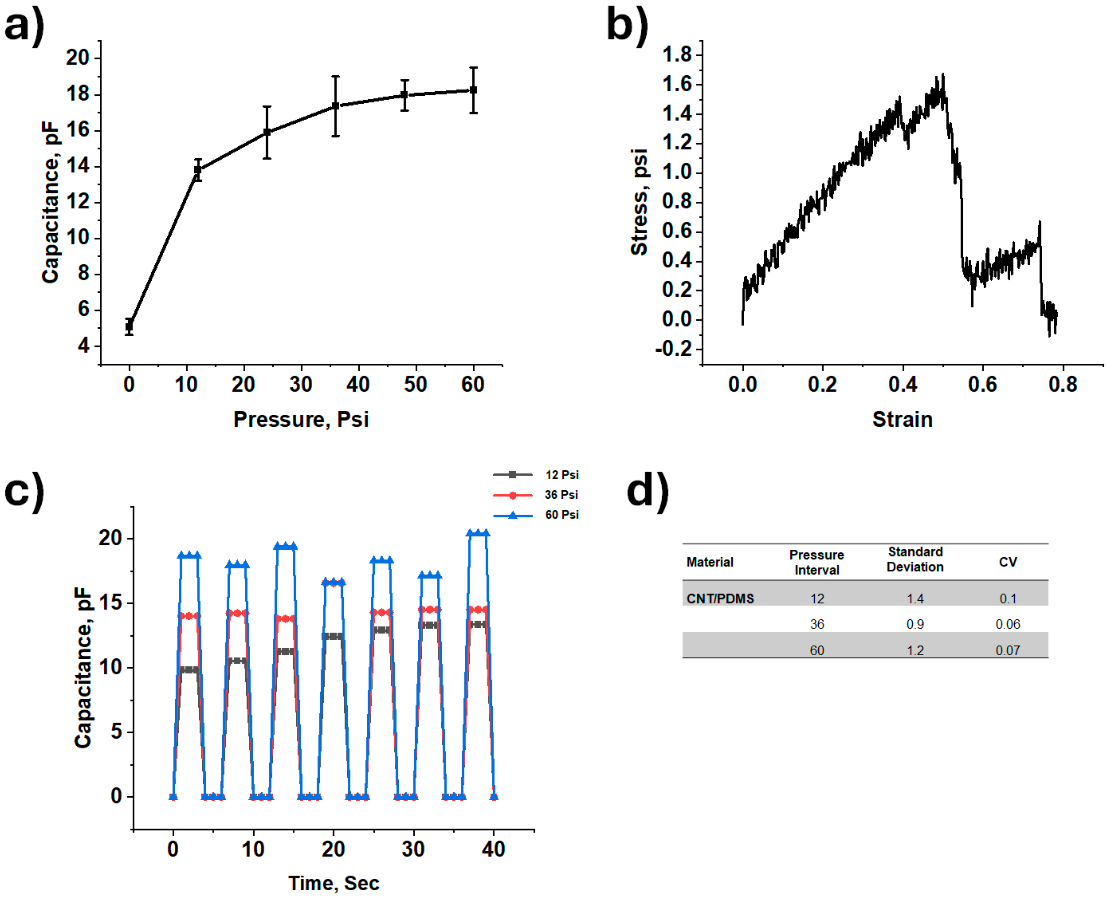

Figure S3. Electrical and mechanical properties of the 3wt% CNT/PDMS (1.1 mm thickness) composite material a) Capacitance vs pressure measurements using applied hand pressure on 3wt% CNT/PDMS (1.1. mm thickness) b) Mechanical testing properties Tensile stress vs strain; c) Mechanical testing properties of 3wt% CNT/PDMS (1.1. mm thickness) Cyclic testing d) Statistical data for cyclic testing of 3wt% CNT/PDMS (1.1 mm thickness)

Figure S3a shows the electrical properties of PDMS/CNT with capacitance measurements upon material under pressure ~18 pF at 20 psi. Figure S3b shows the strain vs. stress graph for the 3 wt% PDMS/CNT composite. The irregularities in the graph are attributed to the improper grips mentioned earlier. Despite this, distinct peaks and drops are observed, with the initial fracture occurring at a stress value of approximately 0.6 psi, labeled as the ultimate tensile strength. The graph indicates that the PDMS/CNT composite exhibited brittle behavior, fracturing at multiple points before a complete break. This behavior is due to the presence of large air pockets, which created a highly porous internal structure in the material. These air pockets led to localized stress concentrations, causing fractures at different loads across the three trials, which showed varying behaviors and ultimate strengths. This variability raises concerns about the repeatability and reliability of the product. However, the size of air bubbles is typically proportional to the mold size, meaning that curing the material in smaller molds could result in a more controlled internal structure and reduce the discrepancies observed in larger molds. Under cyclic loading, PDMS/CNT shows greater fluctuations and higher CV and standard deviation than Ecoflex/CNT, indicating lower reliability, particularly at low pressure as depicted in figure S3c and d

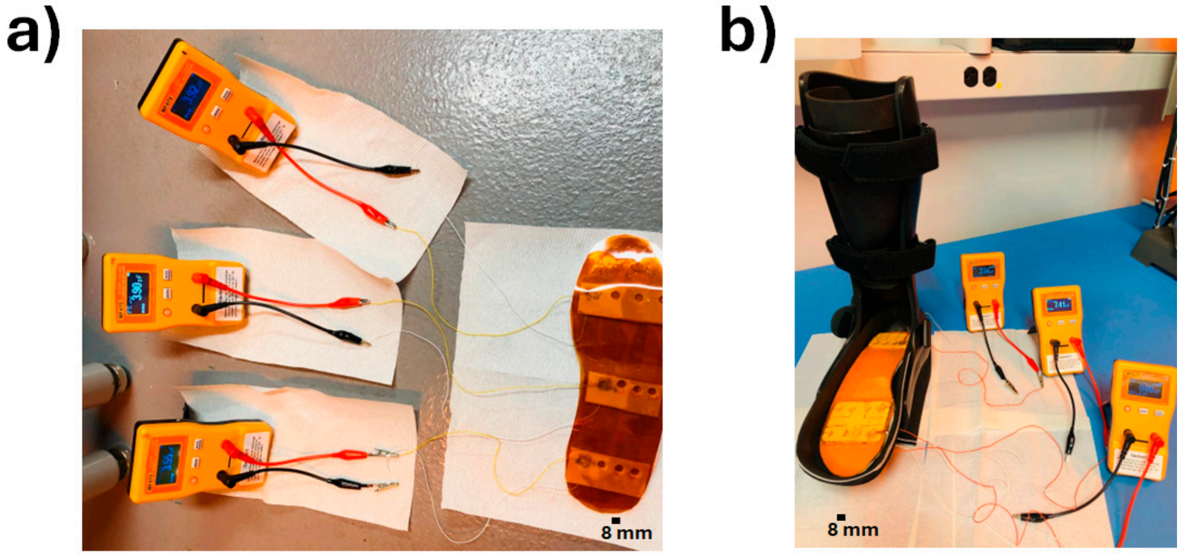

Figure S4 a) Capacitance measurements of the sensor array in the CNT/Ecoflex sensor integrated into the insole boot prototype b) Shows capacitance measurements of the sensor array in the CNT/Ecoflex sensor integrated into the insole boot prototype setup.

Figure S5 shows the stability measurement setup for CNT/Ecoflex single node sensor.

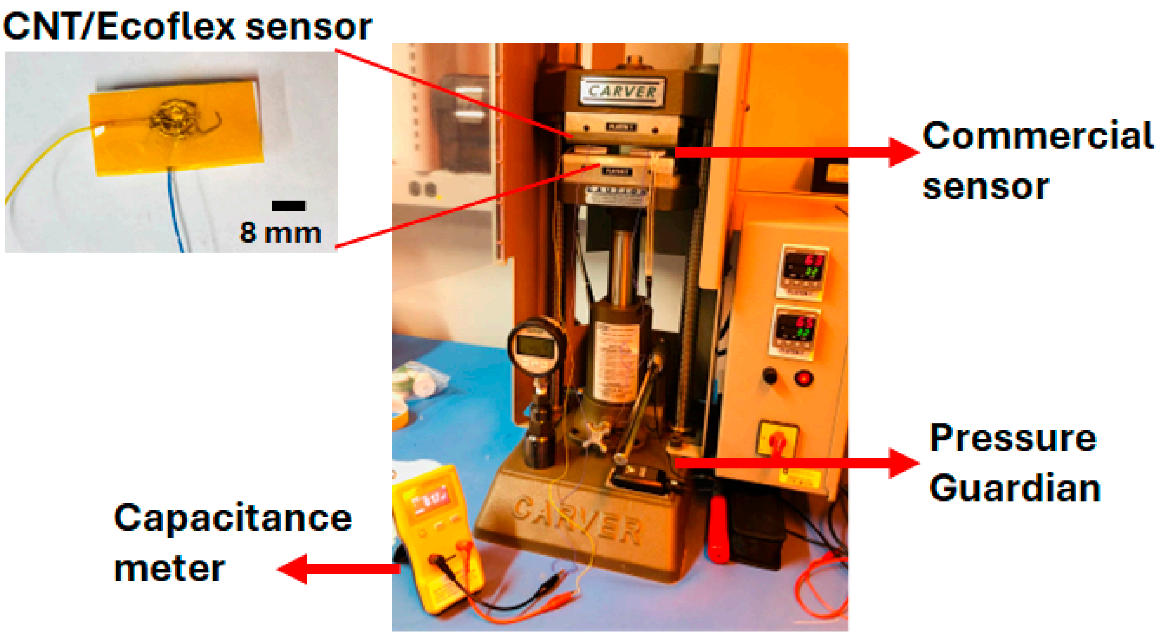

Figure S5 Stability measurement setup for CNT/Ecoflex sensor.
